# Mapping the Energetics of Defect States in Cu_2_ZnSnS_4_ films and the Impact of Sb Doping

**DOI:** 10.1021/acsaem.1c03729

**Published:** 2022-03-22

**Authors:** Devendra Tiwari, Michael V. Yakushev, Tristan Koehler, Mattia Cattelan, Neil Fox, Robert W. Martin, Reiner Klenk, David J. Férmin

**Affiliations:** 1 Department of Mathematics, Physics and Electrical Engineering, Northumbria University, Ellison Place, NE1 8ST Newcastle upon Tyne, United Kingdom; 2 School of Chemistry, University of Bristol, Cantocks Close, BS8 1TS Bristol, United Kingdom; 3 Department of Physics, SUPA, Strathclyde University, G4 0NG Glasgow, United Kingdom; 4 M.N. Miheev Institute of Metal Physics of the UB RAS, 18 S. Kovalevskoy St., 620108 Ekaterinburg, Russia; 5 Ural Federal University, 19 Mira St., 620002 Ekaterinburg, Russia; 6 Institute of Solid-State Chemistry of the UB RAS, 620990 Ekaterinburg, Russia; 7 Faculty of Physics, University of Duisburg-Essen, Forsthausweg 2, 47057 Duisburg, Germany; 8 H. H. Wills Physics Laboratory, University of Bristol, Tyndall Avenue, BS8 1TL Bristol, United Kingdom; 9 Helmholtz-Zentrum Berlin für Materialien und Energie, Hahn-Meitner-Platz 1, D-14109 Berlin, Germany

**Keywords:** Cu_2_ZnSnS_4_ films, defect states, photoluminescence, admittance spectroscopy, quasi-donor−acceptor pairs, photoemission electron microscopy, Sb doping

## Abstract

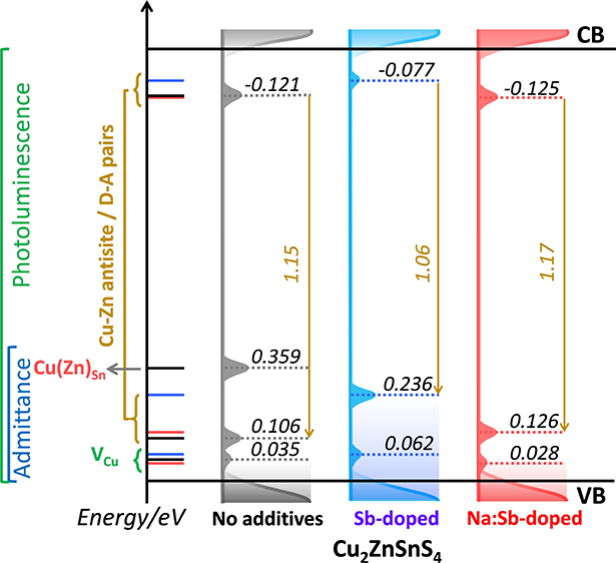

The sub-bandgap levels associated with defect states in Cu_2_ZnSnS_4_ (CZTS) thin films are investigated by correlating the temperature dependence of the absorber photoluminescence (PL) with the device admittance spectroscopy. CZTS thin films are prepared by thermolysis of molecular precursors incorporating chloride salts of the cations and thiourea. Na and Sb are introduced as dopants in the precursor layers to assess their impact on Cu/Zn and Sn site disorder, respectively. Systematic analysis of PL spectra as a function of excitation power and temperature show that radiative recombination is dominated by quasi-donor–acceptor pairs (QDAP) with a maximum between 1.03 and 1.18 eV. It is noteworthy that Sb doping leads to a transition from localized to delocalized QDAP. The activation energies obtained associated with QDAP emission closely correlate with the activation energies of the admittance responses in a temperature range between 150 K and room temperature in films with or without added dopants. Admittance data of CZTS films with no added dopants also have a strong contribution from a deeper state associated with Sn disorder. The ensemble of PL and admittance data, in addition to energy-filtered photoemission of electron microscopy (EF-PEEM), shows a detailed picture of the distribution of sub-bandgap states in CZTS and the impact of doping on their energetics and device performance.

## 1 Introduction

Cu_2_ZnSnS_4_ (CZTS) has tremendous potential as a solar absorber based on its high chemical stability, low toxicity, and optoelectronic properties comparable to Cu(In,Ga)Se_2_ (CIGS) but without critical raw materials.^1,2^ Recent works by Yan et al. have reported efficiencies in the range of 11% for pure sulfide,^3^ while for the partially selenized composition, Son et al. have reported certified power conversion efficiency of 12.62%.^4^ It is widely accepted that the limiting factor in this technology is the significant open-circuit voltage (*V*_OC_) deficit, which is approximately 50% of the Shockley–Queisser limit.^5^ However, the origin of the *V*_OC_ deficit remains to be fully elucidated.

Voltage deficiency in these devices is often linked to the structural disorder in the absorber layer, ranging from secondary phases to intrinsic point defects.^6−8^ Detailed structural analysis under off-stoichiometric composition yielding high-efficiency devices, that is, Cu-poor and Zn-rich, has shown that the primary types of defects are Cu vacancies (V_Cu_), Cu–Zn antisites (Zn_Cu_), and Zn–Sn (Zn_Sn_) disorder.^9^ Such elemental disorder and defects manifest as band edge distortions leading to band tailing and midgap states, restricting the optimal quasi-Fermi level splitting and thus *V*_OC_.^8,10^ Computational studies under ideal thermodynamic equilibrium conditions suggest that clustering of Cu–Zn antisites can lead to band tailing, while Sn-based defects could be responsible for highly detrimental midgap states.^11^

Different synthesis strategies have been proposed to mitigate defects in kesterite thin films, including optimization of precursor composition and thermal annealing conditions.^12^ However, doping and alloying have emerged as a key approach to minimize elemental disorder.^2^ The introduction of alkali cations and Ag^+^ has been widely investigated to reduce disorder on Cu^+^ sites, while doping or substitution with Cd has also been investigated toward mitigating disorder on Zn sites.^5,13^ With regards to the Sn site, one of the strategies implemented so far is Ge doping or alloying.^2,5^

Our previous studies have shown that Sb, which has been used for recrystallization reflux in CIGS growth,^14^ can lead to a decrease in Sn disorder and improvement of CZTS device efficiency.^15,16^ Indeed, analysis of more than 200 devices has shown that Na:Sb co-doping lead to a 60 mV increase in *V*_OC_, 10% increase in fill factor (FF), and an overall power conversion efficiency (η) gain of more than 1.5% with respect to nondoped materials.^16^ We have observed that the Sb distribution across the CZTS film thickness is inhomogeneous. Na co-doping assists in regulating the Sb uptake along with significantly modifying the surface electronic landscape of the films.^15^ In this work, we elucidate the impact of Na and Sb doping on the distribution of sub-bandgap states associated with the elemental disorder in CZTS thin films by examining the temperature dependence of the photoluminescence (PL) spectrum of CZTS thin films and the temperature dependence of the device admittance. This approach allows correlating chemically specific signatures from PL measurements of the absorber layer to device admittance responses. PL responses are dominated by quasi-donor–acceptor pairs (QDAPs), which have a localized or a nonlocalized nature depending on the dopant. We conclude that the dynamics of populating and depopulating states associated with QDAP in doped absorbers dominates the device admittance at temperatures above 150 K, while CZTS with no added dopants features a deeper sub-bandgap state, which is linked to Sn disorder.

## 2 Results and Discussion

### 2.1 Temperature-Dependent Photoluminescence (PL) Measurements

CZTS films were generated, as described in the Experimental section, by spin-coating of a molecular precursor containing chloride salts of the various cations and thiourea dissolved in a mixture of dimethyl-formamide and isopropyl alcohol, followed by annealing under Ar at 560 °C for 30 min.^16^ Dopants were also introduced directly in the precursor solution as metal salts. In this study, the films obtained without any additional dopants in the precursor solution will be labeled “no dopants” (ND), while those obtained upon adding antimony acetate are labeled “Sb-doped”, and the films obtained with coaddition of antimony acetate and sodium chloride are called “Na:Sb co-doped”. Mo-coated glass in this study was procured from a vendor fabricating these substrates for a commercial CIGS module producer. In our analysis of annealed ND CZTS films, we could not detect any Na through secondary ion mass spectrometry (SIMS) depth profile, energy dispersive X-ray spectroscopy (EDS), and X-ray photoelectron spectroscopy (XPS) of the front CZTS surface or wavelength dispersive X-ray spectroscopy (WDS) of exposed CZTS from the CZTS/Mo interface.^15^ Additionally, our previous attempts at sole addition of NaCl to the precursor solution did not lead to any substantial improvement of device performance.^16^

**Figure 1:**
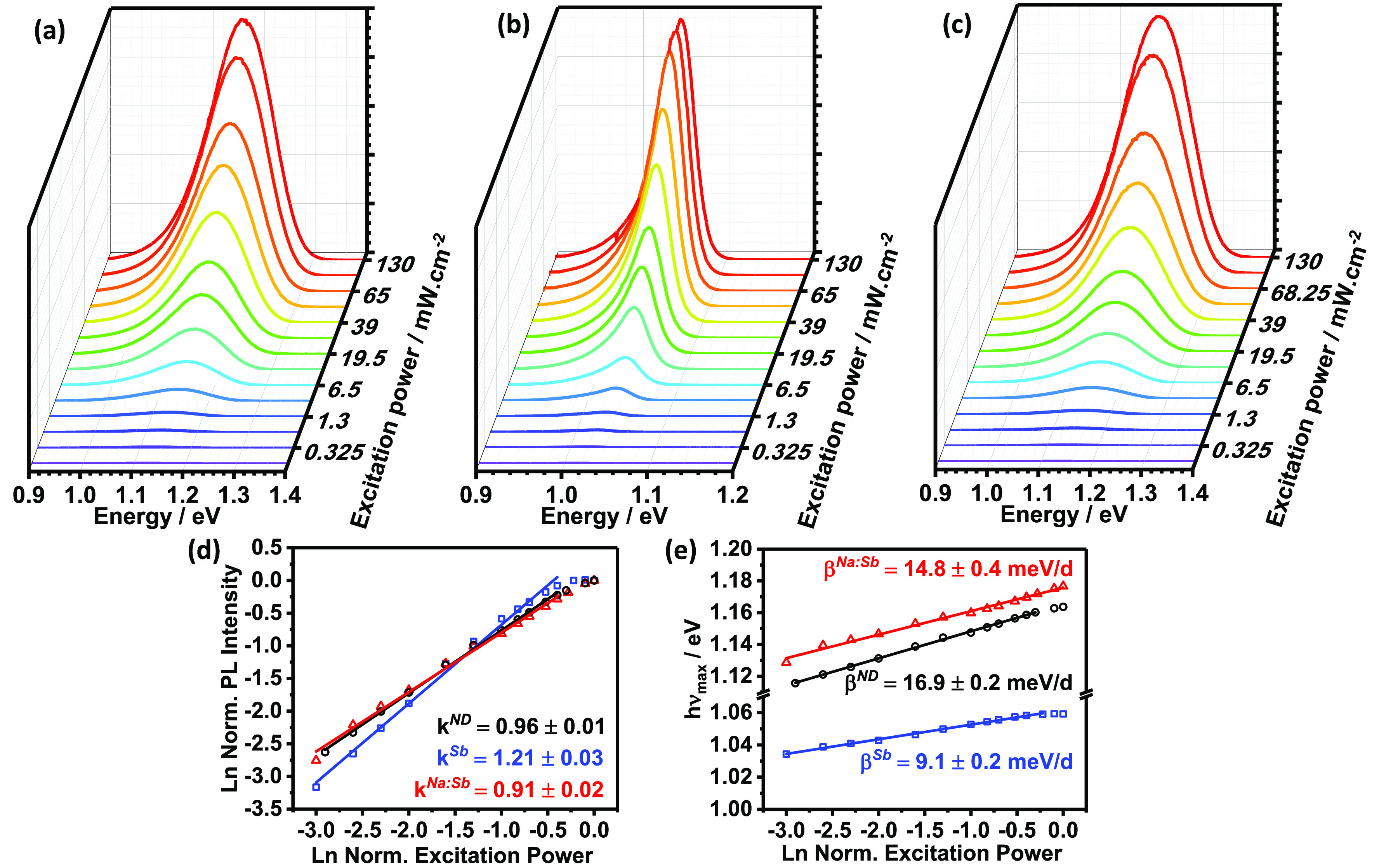
Excitation power dependence of the photoluminescence (PL) spectra of CZTS films at 5 K: ND (a), Sb-doped (b), and Na:Sb co-doped (c). Changes in normalized PL intensity (d) and PL maximum (e) as a function of changes in excitation power at 5 K. The experimental trends show that the PL transitions are dominated by radiative recombination via quasi-donor–acceptor pairs (QDAPs) of a localized nature in the case of ND and Na:Sb co-doped CZTS, while a more delocalized character is observed in Sb-doped films.

Photoluminescence (PL) spectra of CZTS films as a function of excitation laser power at 5 K are shown in Figure 1. All spectra exhibit a broad asymmetric photoluminescence band with a maximum located between 1.03 and 1.18 eV, depending on laser power, temperature, and extrinsic doping. Interestingly, the PL band of the Sb-doped film is significantly narrower (Figure 1b) in comparison to the ND (Figure 1a) and Na:Sb co-doped (Figure 1c) CZTS films. To rationalize the PL trends, we have fitted the spectra to a double sigmoidal function (DSF) in the range of 0.9 and 1.3 eV (section 4). The DSF was implemented by Krustok et al. to investigate disordered chalcogenides featuring band tailing.^17^

The dependence of the primary PL peak intensity with excitation laser power is shown in Figure 1d, which is fitted to the power law
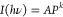
1where *I* is the PL peak intensity, *A* is a proportionality constant, *P* is excitation power, and the exponent *k* is a parameter associated with the recombination mechanism.^18^ Values of *k* obtained for ND and Na:Sb doped were below 1, indicative of *localized* defect mediated transitions.^19−24^ On the other hand, Sb-doped films exhibit *k* = 1.2, which strongly suggests a *nonlocalized* defect as reported in other studies involving chalcopyrites and kesterites.^19−24^ This shift from localized to nonlocalized defect mediated transitions is an indication that the positions of the states involved in the PL transition are shifted with respect to the band edges depending on the Sb content.

Figure 1e shows a strong blue shift (β) of the PL maximum between 9 and 17 meV per decade of laser power, which has been linked to radiative recombination via quasi-donor–acceptor pairs (QDAPs).^20,24−26^ This radiative mechanism is slightly different from the process observed for classical donor–acceptor pairs in which the electrostatic term, responsible for the blue shift with laser power, generates significantly weaker dependence.^20,24,27^ The QDAP model additionally includes potential energy fluctuation associated with elemental disorder, which can be expressed as
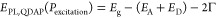
2where *E*_g_, *E*_A_, *E*_D_, and Γ are the bandgap, acceptor and donor energy level positions, and the average depth of the potential energy fluctuations, respectively. The blue shift observed with increasing excitation power results from the emptying of tail states and screening of the potential fluctuations by the photogenerated carriers. In our analysis, Γ values are extracted from the DSF fitting of the PL spectra (see section 4), yielding values of 35, 18, and 22 meV for the ND, Sb-doped, and Na:Sb-doped CZTS films at 5 K, respectively.

**Figure 2:**
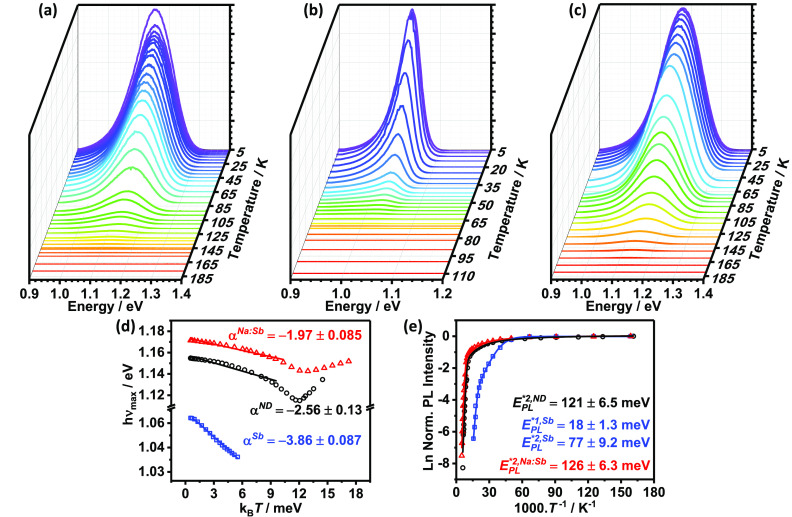
Temperature dependence of PL spectra of CZTS films: ND (a), Sb-doped (b), and Na:Sb co-doped (c). Variation of PL maximum (d) and intensity (e) with temperature. The α value in panel d is inversely proportional to the concentration of majority carriers.

The temperature dependence of the PL responses for all three samples is shown in Figure 2a–c. The PL intensity of the ND and Na:Sb co-doped samples displays a weaker decay with increasing temperature than that of the Sb-doped films. Figure 2d shows the temperature dependence of the peak position switching from bathochromic to hypsochromic behavior at temperatures >140 K. This observation further supports the QDAP radiative recombination model, in which increasing the temperature leads to population and depopulation of the impurity levels and tail states, respectively, which is responsible for the change in temperature dependence at 140 K.^28^ The magnitude of the red shift as indicated by the slope (α) of the linear portion is much larger than the reported temperature-dependent bandgap narrowing.^29^ Levanyuk and Osipov’s model for disordered semiconductors proposes an inverse dependence of α with doping concentration,^30^
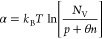
3where *N*_V_ is the effective density of states at the valence band, θ is the ratio of the electron and hole capture cross sections, and *p* and *n* are the hole and electron concentrations, respectively. According to the trends in Figure 2d, the Sb-doped films have a lower carrier concentration than the ND and Na:Sb co-doped materials.

Figure 2e depicts the dependence of the integrated PL intensity with the inverse of temperature for all three samples. In the case of the ND and Na:Sb-doped CZTS, which are characterized by emission from localized states (Figure 1d), the thermal quenching of the PL intensity can be described by a single recombination pathway with a temperature-dependent cross-section:^31^
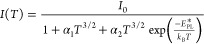
4where *I*_0_ is the PL intensity at 5 K, α_1_ and α_2_ determine the temperature dependence of the capture cross-section and *E*_PL_^*^ is the activation energy. The analysis in Figure 2e shows a good fit to this model with *E*_PL_^*^ values of 121 ± 6 meV and 126 ± 9 meV for ND and Na:Sb co-doped films, respectively. In the case of Sb-doped films, the nonlocalized nature of the radiative recombination states can be rationalized by an Arrhenius type model with two exponential terms:
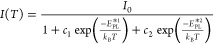
5where *c*_1_ and *c*_2_ are the corresponding pre-exponential terms. The activation energies obtained from this analysis are 18 ± 1 meV and 77 ± 9 meV.

**Figure 3:**
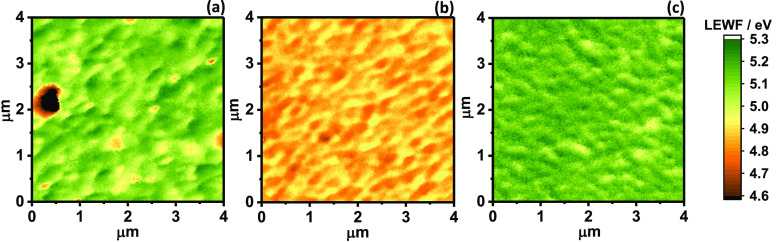
Local effective work function (LEWF) maps constructed from the energy-filtered photoemission electron microscopy (EF-PEEM) of ND (a), Sb-doped (b), and Na:Sb co-doped (c) CZTS films. The mean LEWF values across the films are approximately 5.1 eV for ND and Na:Sb co-doped CZTS, while Sb doping leads to a mean value of 4.8 eV.

The temperature and excitation power dependences of the PL reveal very similar features for the ND and Na:Sb co-doped films and are very distinct from those of the Sb-doped films, which demonstrates a significant contrast in the electronic properties of the semiconductor thin films. This is further illustrated in the local effective work function maps in Figure 3, obtained from energy-filtered photoemission electron microscopy (EF-PEEM). In agreement with previous studies,^15^ we can observe that the mean effective work function of the Sb-doped films is 0.3 eV lower than for ND and Na:Sb co-doped films. As a group V cation, Sb^3+^ is expected to act as an electron-donating state upon substituting Sn^4+^ cation in CZTS, which leads to charge compensation effects that lowers the hole concentration (majority carrier). As we demonstrate further below, the lowering of the work function caused by Sb doping leads to a partial overlap of the donor state and the conduction band edge energies, which is responsible for the nonlocalized nature of the radiative recombination states and the significantly different PL line shape. On the other hand, the introduction of Na^+^ not only leads to an increase of majority carrier concentration^5^ but also regulates the uptake of Sb in the film,^15,16^ which manifests itself by an increase of the mean effective work function to values close to 5.1 eV. As a result, the QDAP recovers their localized nature, and the PL responses adopt similar behavior to the ND samples.

It is also noticeable that ND CZTS films, with no added Sb, show regions of low work function values in the range 4.6 to 4.8 eV, as exemplified in Figure 3a. In a previous study, we examined the valence band spectra of similar work function regions in CZTSSe films, which were consistent with surface confined Sn(II) chalcogenide phases.^32^ These low work function regions are not observed in Sb-doped and Na:Sb co-doped films, further suggesting that Sb does play a role in minimizing Sn disorder. As discussed further below, this effect has a clear impact on device performance.

### 2.2 Temperature-Dependent Admittance Spectroscopy

**Figure 4:**
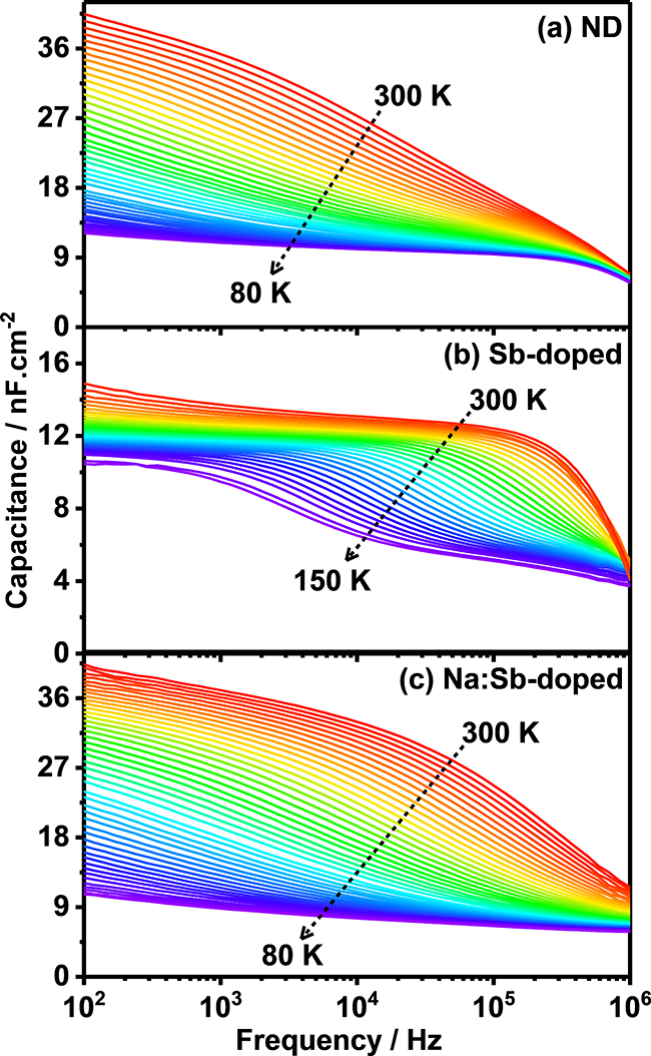
Frequency dependence of the device capacitance between 80 and 300 K, featuring ND (a), Sb-doped (b), and Na:Sb co-doped CZTS films (c). The capacitance was estimated from the imaginary component of the admittance responses under 10 mV RMS sinusoidal potential perturbation around the equilibrium potential of the device in the dark. The power conversion efficiencies of the devices were 4.15, 4.99, 5.60%, respectively (see Figure S1).

The capacitance values as a function of frequency for three devices based on ND, Sb-doped, and Na:Sb-codoped CZTS films, in the temperature range between 80 and 300 K, are displayed in Figure 4. Details of device preparation are included in section 4, while current–voltage characteristics and external quantum efficiency spectra are displayed in Figure S1 and Figure S2 (Supporting Information). As summarized in Table S1, all mean values of *J*_SC_, *V*_OC_, FF, and PCE obtained from 72 devices of each formulation increase upon Sb-doping and Na:Sb co-doping. A more detailed analysis of the evolution of device characteristics upon doping is published elsewhere.^16^ The capacitance values are calculated from the imaginary component of the admittance recorded using a 10 mV rms potential perturbation at the device equilibrium potential in the dark. Consequently, these responses reflect the dynamics of population and depopulation of states near the Fermi level, which is located close to the valence band edge. Comparing the three sets of data in Figure 4, it can be clearly seen that the capacitance of Sb-doped CZTS devices is smaller than that of ND and Na:Sb co-doped thin films. This observation is entirely consistent with our analysis of the temperature dependence of the PL intensity (Figure 2d), which indicated that the density of majority carriers (holes) in the Sb-doped films was smaller than that in ND and Na:Sb co-doped films. This observation also qualitatively agrees with the lower work function values observed in Sb-doped films (Figure 3). The low hole concentration in Sb-doped films also manifests itself in an early carrier freeze-out at 150 K, which is 70 K higher than the that of other CZTS devices.

**Figure 5:**
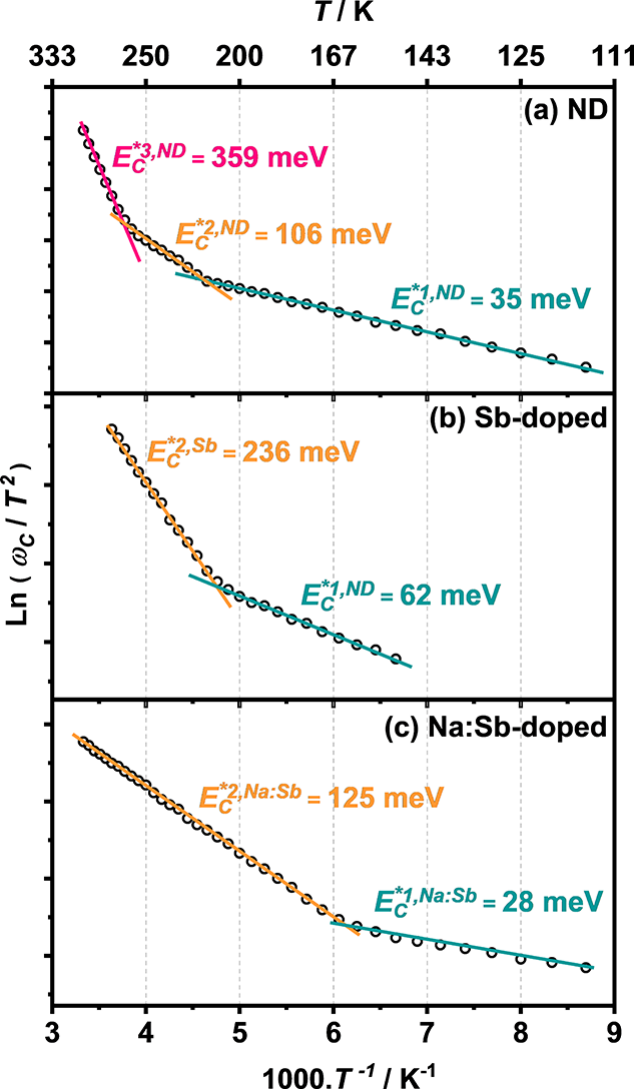
Arrhenius plots of the characteristic frequency (ω_c_) associated with population or depopulation of defects states obtained from admittance data as a function of temperature: ND (a), Sb-doped (b), and Na:Sb co-doped CZTS devices (c).

Following the analysis reported by Walter et al.,^33^ plotting *f* vs  leads to a series of inflection points at a characteristic frequency (ω_c_), which are associated with population and depopulation dynamics of trap states. The activation energy (*E*_C_^*^) associated with these dynamic processes can be calculated from the plot of *T* vs  as illustrated in Figure 5. ND CZTS devices (Figure 5a) show three different activation energy values operating in three temperatures ranges: between 110 and 200 K *E*_C_^*1,ND^ = 35 meV, from 200 to 265 K *E*_C_^*2,ND^ = 106 meV, and from 265 to 300 K *E*_C_^*3,ND^ = 359 meV. Interestingly, Na:Sb co-doped CZTS devices (Figure 5c) show a similar trend with *E*_C_^*1,Na:Sb^ = 28 meV and *E*_C_^*2,Na:Sb^ = 125 meV. Neither Sb-doped nor Na:Sb co-doped CZTS devices show evidence of the deeper state observed in ND devices (*E*_C_^*3,ND^). The Sb-doped CZTS devices (Figure 5b) show the two activation steps *E*_C_^*1,Sb^ = 62 meV and *E*_C_^*2,Sb^ = 236 meV at energies relatively higher than in the case of ND and Na:Sb co-doped devices. The shift in the activation steps in Sb-doped devices is another clear manifestation of the lower work function of the absorber layer (Figure 3), generating a higher energy difference between the Fermi energy and the associated defect states.

### 2.3 Energetics of Defect States Arising from PL and Admittance Spectroscopy

**Figure 6:**
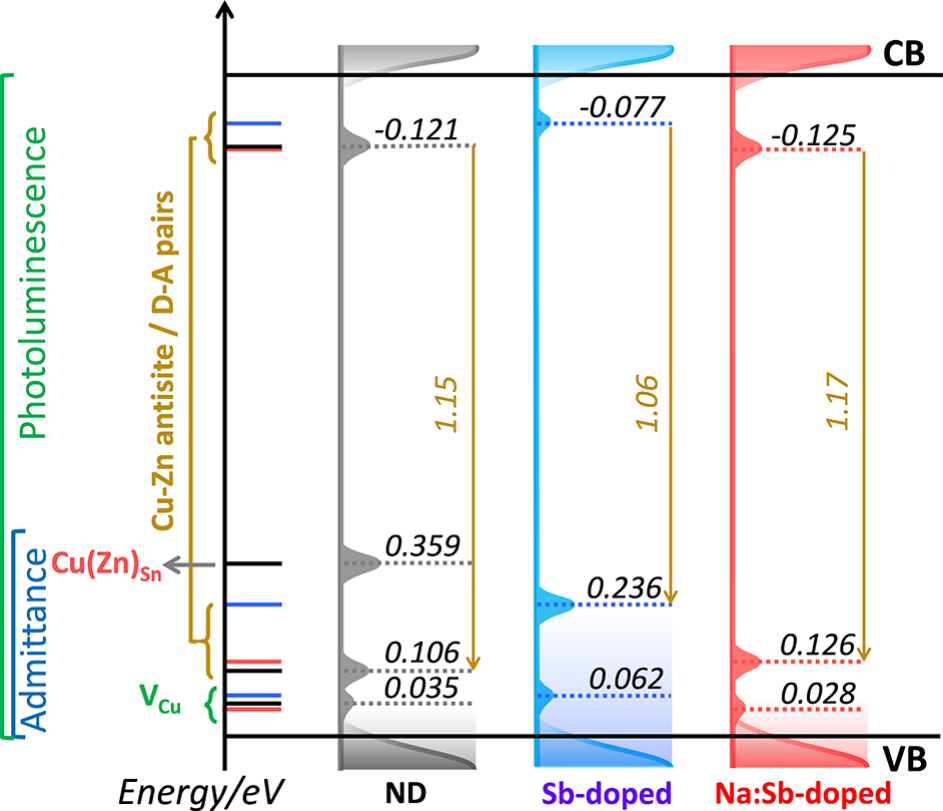
Energetics of defect states in CZTS and the impact of Sb and Na:Sb co-doping as probed by temperature-dependent PL of the absorber layer and device admittance spectroscopy. Energy levels in each diagram are referenced to same valence band edge energy. Consequently, changes in work function upon doping are represented as a shift in the position of the sub-bandgap states. Energy values are presented in eV. The sum of the energy of radiative transition, QDAP energy levels, and average depth potential energy fluctuations for both band edges is the same as bandgap of 1.43 eV as probed by external quantum efficiency spectra (Figure S3, Table S2).

The close correspondence between the activation energies of PL spectra of the thin films and device admittance spectra allows building a consistent picture of the energetics of key defect states, as illustrated in Figure 6. In this representation, the valence band edge is used as the reference energy; therefore, the observed changes in work function are represented as shifts of the sub-bandgap states. Table S2 summarizes how each of these states were estimated from the various experimental methods. It should be noted that the relative position of the sub-bandgap states estimated from PL and admittance spectroscopy are consistent with the bandgap of the material, all obtained from independent measurements.

The shallower states identified as *E*_C_^*1^ can be linked to Cu vacancies (V_Cu_), which is consistent with a variety of experimental and computational studies, which estimated transition energies in the range of 15 to 70 meV.^21,24,34,35^ The close correspondence between *E*_PL_^*^ and *E*_C_^*2^ values suggest that these parameters have a common origin, with Cu_Zn_ clusters being the most likely defect. Indeed, the energetics of this defect are also consistent with computational studies of point defects.^34,35^ It should also be mentioned that the deeper state probed by the admittance spectra of the ND absorber, *E*_C_^*3,ND^, is very likely to be associated with Sn disorder, for example, Cu_Sn_ or Zn_Sn_ defects. This is consistent with the reduction of low work function regions (shunts), most probably linked to Sn(II)S, as probed by EF-PEEM (Figure 3). A more quantitative analysis of the Sb doping on the surface electronic landscape has been published elsewhere.^15^

Finally, the impact of the dopants on the electronic structure of CZTS translates to changes in device performance as illustrated in Figure S1 and Table S1. The systematic increase in mean PCE values from 3.21 ± 0.64 (ND) to 4.70 ± 0.29 (Sb-doped) to 5.04% ± 0.35% (Na:Sb co-doped) demonstrates that the evolution of the defect levels illustrated in Figure 6 generates statistically sound improvements in every device performance metric. We acknowledge that the champion device PCE efficiency of Na:Sb co-doped films, 5.72% under AM1.5 illumination, remains significantly lower than the 11.1% record efficiency reported from optimized sputtering methods.^3^ Although optimization of parameters such as CdS thickness (which significantly limits our device current outputs) will lead to important substantial PCE efficiency, there is much room for further optimization of precursor composition and dopant inclusion, which could be designed based on the conclusions of this report.

## 3 Conclusions

Detailed analysis of the temperature dependence of the PL spectra of CZTS thin films and the device admittance spectroscopy and the impact of Sb and Na:Sb co-doping allows establishment of the energetics of key sub-bandgap states, which determine device properties. The spectral responses are dominated by QDAP radiative recombination involving localized states, except in Sb-doped CZTS, in which changes in the work function of the material leads to a close interaction between the donor state and the conduction band. Activation energy terms obtained from the PL responses of the film and the admittance responses of the devices allows establishing a self-consistent picture of the energetics of defect states, including V_Cu_, Cu_Zn_, and Cu(Zn)_Sn_, which bodes well with the computational studies of point defects in these complex materials. These observations are extremely valuable for developing diagnostic criteria for generating high-efficiency CZTS solar cells. Given that Cu_Zn_ and Zn_Cu_ defects are ubiquitous to this class of materials, even upon extrinsic doping, it is very likely that the maximum *V*_OC_ available for these devices, as is determined by the energies of the QDAP transitions, will be limited to 1.1 V, that is, ∼75% of the bandgap. However, this value is still significantly higher than the current record devices (0.73 V)^3^; therefore, there is significant room for further improvement in device performance.

## 4 Experimental Section

We have previously reported the complete film deposition and device completion protocol.^16^ ND, Sb-doped, and Na:Sb co-doped CZTS films are deposited by spin-coating a single solution precursor onto a 5 × 5 cm^2^ Mo-coated glass substrate (MSolv, U.K.) thermally pretreated at 300 °C in air. This step is repeated four times to attain film thickness of 1.2 μm. The precursor solution is composed of dimethylformamide and isopropanol (DMF–IPA) containing metal chloride salts and thiourea. The dopants were introduced in the same precursor solution by additionally adding antimony(III) acetate (1 atom %) and sodium chloride salts (0.2 atom %). For annealing, the films were placed in graphite boxes with S powder and heated at 560 °C for 30 min in a rapid thermal annealing furnace (MTI-OTF1200X) under Ar atmosphere.

The devices with architecture SLG/Mo/CZTS/CdS/i-ZnO/ZnO:Al/Ni–Al were fabricated from the annealed films by first etching CZTS in a 10% aqueous KCN solution for 30 s. This step was immediately followed by chemical bath deposition of a CdS layer at 70 °C from an aqueous bath consisting of CdSO_4_, NH_4_OH, and thiourea.^16^ The devices were completed by depositing i-ZnO and Al:ZnO transparent conducting oxide layers by RF-sputtering, followed by evaporation of Ni–Al top contacts through a shadow mask, with no antireflection coating used. The devices were mechanically scribed to have a total area of 0.5 cm^2^.

The device performance was measured through *J*–*V* characteristics under dark and under simulated AM1.5G (100 mW/cm^2^) illumination from a class AAA solar simulator. No aperture masks were used. The external quantum efficiency (EQE) was measured using a custom-configured spectrometer composed of a dual halogen–xenon lamp source and a Bentham instruments TM 300 monochromator. The PV measurement set-ups were calibrated with reference cells or Si and Ge photodiodes from Newport Corporation.

The details of energy-filtered photoemission electron microscopy (EF-PEEM) were elaborated in our previous work, and EF-PEEM was performed at the Bristol NanoESCA facility.^15^ In this work, EF-PEEM had a nominal spatial resolution of 150 nm and was acquired using a He−Ι (21.2 eV) light source. The sample was held at 1.8 mm from the extractor kept at 12 kV. During the room-temperature EF-PEEM scans, an entrance slit of 0.5 mm and a pass energy of 50 eV were employed, giving an overall energy resolution of 140 meV, estimated from a clean Fermi edge estimation of a clean metallic substrate.

Capacitance data was calculated from admittance spectra measured using a Modulab impedance analyzer in the frequency range of 1 Hz and 1 MHz with an AC stimulus of 10 mV root mean square (rms) in a modified Linkam HFS 600PB4 variable temperature cell at temperature steps of 5 K in the cooling cycle.

Photoluminescence spectra were measured with custom-built spectrometer, including a 514.5 nm Ar-ion laser as excitation source, while spectral acquisition was performed through a Hilgar–Watts monochromator with 1 m focal length and a biased InGaAs photodetector for acquiring the spectrum. An Advanced Research Systems closed-cycle liquid helium cryostat was used for temperature regulation.

The PL spectra were fitted with the double sigmoidal function proposed by Krustok et al.^31^
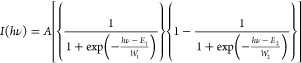
6where parameters *A*, *E*, and *W* are the peak area, position, and width, respectively. *E*_1_ and *W*_1_ correspond to the lower energy side of the PL peak.

## Supporting Information Available

*J*–*V* characteristics of solar cells investigated under dark and AM1.5G illumination, EQE spectral response of the studied devices and plot to determine bandgap from EQE spectrum, statistical analysis of solar cell performance metrics, and tabulated summary of defect states and optoelectronic parameters from EQE, admittance, and PL spectroscopies (PDF)

## Notes

All data presented in this research article can be freely accessed from the Northumbria University’s Research Data Repository at https://doi.org/10.25398/rd.northumbria.19209678.v1.

## References

[ref1] European Commission. List of Critical Raw Materials for the EU; 2017.

[ref2] Giraldo S.; Jehl Z.; Placidi M.; Izquierdo-Roca V.; Pérez-Rodríguez A.; Saucedo E. Progress and Perspectives of Thin Film Kesterite Photovoltaic Technology: A Critical Review. Adv. Mater. 2019, 31, 1806692 10.1002/adma.201806692. 30767308

[ref3] Yan C.; Huang J.; Sun K.; Johnston S.; Zhang Y.; Sun H.; Pu A.; He M.; Liu F.; Eder K.; Yang L.; Cairney J. M.; Ekins-Daukes N. J.; Hameiri Z.; Stride J. A.; Chen S.; Green M. A.; Hao X. Cu_2_ZnSnS_4_ Solar Cells with over 10% Power Conversion Efficiency Enabled by Heterojunction Heat Treatment. Nat. Energy 2018, 3, 764–772. 10.1038/s41560-018-0206-0.

[ref4] Son D.-H.; Kim S.-H.; Kim S.-Y.; Kim Y.-I.; Sim J.-H.; Park S.-N.; Jeon D.-H.; Hwang D.-K.; Sung S.-J.; Kang J.-K.; Yang K.-J.; Kim D.-H. Effect of Solid-H_2_S Gas Reactions on CZTSSe Thin Film Growth and Photovoltaic Properties of a 12.62% Efficiency Device. J. Mater. Chem. A 2019, 7, 25279–25289. 10.1039/C9TA08310C.

[ref5] Romanyuk Y. E.; Haass S. G.; Giraldo S.; Placidi M.; Tiwari D.; Fermin D. J.; Hao X.; Xin H.; Schnabel T.; Kauk-Kuusik M.; Pistor P.; Lie S.; Wong L. H. Doping and Alloying of Kesterites. J. Phys. Energy 2019, 1, 044004 10.1088/2515-7655/ab23bc.

[ref6] Platzer-Björkman C.; Scragg J.; Flammersberger H.; Kubart T.; Edoff M. Influence of Precursor Sulfur Content on Film Formation and Compositional Changes in Cu_2_ZnSnS_4_ Films and Solar Cells. Sol. Energy Mater. Sol. Cells 2012, 98, 110–117. 10.1016/j.solmat.2011.10.019.

[ref7] Lafond A.; Choubrac L.; Guillot-Deudon C.; Deniard P.; Jobic S. Crystal Structures of Photovoltaic Chalcogenides, an Intricate Puzzle to Solve: The Cases of CIGSe and CZTS Materials. Zeitschrift fur Anorg. und Allg. Chemie 2012, 638, 2571–2577. 10.1002/zaac.201200279.

[ref8] Siebentritt S.; Schorr S. Kesterites—a Challenging Material for Solar Cells. Prog. Photovoltaics 2012, 20, 512 10.1002/pip.2156.

[ref9] Hood S. N.; Walsh A.; Persson C.; Iordanidou K.; Huang D.; Kumar M.; Jehl Z.; Courel M.; Lauwaert J.; Lee S. Status of Materials and Device Modelling for Kesterite Solar Cells. J. Phys. Energy 2019, 1, 042004 10.1088/2515-7655/ab2dda.

[ref10] Bourdais S.; Chone C.; Delatouche B.; Jacob A.; Larramona G.; Moisan C.; Lafond A.; Donatini F.; Rey G.; Siebentritt S.; Walsh A.; Dennler G. Is the Cu/Zn Disorder the Main Culprit for the Voltage Deficit in Kesterite Solar Cells?. Adv. Energy Mater. 2016, 6, 1502276 10.1002/aenm.201502276.

[ref11] Schorr S.; Gurieva G.; Guc M.; Dimitrievska M.; Pérez-Rodríguez A.; Izquierdo-Roca V.; Schnohr C. S.; Kim J.; Jo W.; Merino J. M. Point Defects, Compositional Fluctuations, and Secondary Phases in Non-Stoichiometric Kesterites. J. Phys. Energy 2020, 2, 012002 10.1088/2515-7655/ab4a25.

[ref12] Ratz T.; Brammertz G.; Caballero R.; León M.; Canulescu S.; Schou J.; Gütay L.; Pareek D.; Taskesen T.; Kim D.-H.; Kang J.-K.; Malerba C.; Redinger A.; Saucedo E.; Shin B.; Tampo H.; Timmo K.; Nguyen N. D.; Vermang B. Physical Routes for the Synthesis of Kesterite. J. Phys. Energy 2019, 1, 042003 10.1088/2515-7655/ab281c.

[ref13] Cherns D.; Griffiths I. J.; Jones L.; Bishop D. M.; Lloyd M. A.; McCandless B. E. Direct Observation of High Densities of Antisite Defects in Ag_2_ZnSnSe_4_. ACS Appl. Energy Mater. 2018, 1, 6260–6267. 10.1021/acsaem.8b01274.

[ref14] Yuan M.; Mitzi D. B.; Liu W.; Kellock A. J.; Chey S. J.; Deline V. R. Optimization of CIGS-Based PV Device through Antimony Doping. Chem. Mater. 2010, 22, 285–287. 10.1021/cm903428f.

[ref15] Tiwari D.; Cattelan M.; Harniman R. L.; Sarua A.; Fox N.; Koehler T.; Klenk R.; Fermin D. J. Impact of Sb and Na Doping on the Surface Electronic Landscape of Cu_2_ZnSnS_4_ Thin Films. ACS Energy Lett. 2018, 3, 2977–2982. 10.1021/acsenergylett.8b02081.

[ref16] Tiwari D.; Koehler T.; Lin X.; Harniman R.; Griffiths I.; Wang L.; Cherns D.; Klenk R.; Fermin D. J. Cu_2_ZnSnS_4_ Thin-Films Generated from a Single Solution Based Precursor: The Effect of Na and Sb Doping. Chem. Mater. 2016, 28, 4991–4997. 10.1021/acs.chemmater.6b01499.

[ref17] Krustok J.; Collan H.; Yakushev M.; Hjelt K. The Role of Spatial Potential Fluctuations in the Shape of the PL Bands of Multinary Semiconductor Compounds. Phys. Scr. 1999, T79, 179–182. 10.1238/Physica.Topical.079a00179.

[ref18] Schmidt T.; Lischka K.; Zulehner W. Excitation-Power Dependence of the near-Band-Edge Photoluminescence of Semiconductors. Phys. Rev. B 1992, 45, 8989–8994. 10.1103/PhysRevB.45.8989. 10000759

[ref19] Zubiaga A.; García J. A.; Plazaola F.; Muñoz-Sanjosé V.; Martínez-Tomás C. Near Band Edge Recombination Mechanisms in GaTe. Phys. Rev. B - Condens. Matter Mater. Phys. 2003, 68, 245202 10.1103/PhysRevB.68.245202.

[ref20] Toginho Filho D. O.; Dias I. F. L.; Laureto E.; Duarte J. L.; Lourenço S. A.; Poças L. C.; Prabhu S. S.; Klem J. Quasi-Donor-Acceptor Pair Transitions in GaAsSb and AlGaAsSb on InP. J. Appl. Phys. 2005, 97, 123702 10.1063/1.1923588.

[ref21] Krustok J.; Raadik T.; Grossberg M.; Trifilleti V.; Binetti S.; et al. Photoluminescence Study of Deep Donor-Deep Acceptor Pairs in Cu_2_ZnSnS_4_. Mater. Sci. Semicond. Process. 2018, 80, 52–55. 10.1016/j.mssp.2018.02.025.

[ref22] Tiwari D.; Skidchenko E.; Bowers J.; Yakushev M. V.; Martin R.; Fermin D. J. Spectroscopic and Electrical Signatures of Acceptor States in Solution Processed Cu_2_ZnSn(S, Se)_4_ Solar Cells. J. Mater. Chem. C 2017, 5, 12720–12727. 10.1039/C7TC03953K.

[ref23] Yakushev M. V.; Sulimov M. A.; Márquez-Prieto J.; Forbes I.; Edwards P. R.; Zhivulko V. D.; Borodavchenko O. M.; Mudryi A. V.; Krustok J.; Martin R. W. A Luminescence Study of Cu_2_ZnSnSe_4_/Mo/Glass Films and Solar Cells with near Stoichiometric Copper Content. J. Phys. D. Appl. Phys. 2019, 52, 055502 10.1088/1361-6463/aaefe3.

[ref24] Levcenko S.; Just J.; Redinger A.; Larramona G.; Bourdais S.; Dennler G.; Jacob A.; Unold T. Deep Defects in Cu_2_ZnSn (S, Se)_4_ Solar Cells with Varying Se Content. Phys. Rev. Appl. 2016, 5, 024004 10.1103/PhysRevApplied.5.024004.

[ref25] Tai K. F.; Gershon T.; Gunawan O.; Huan C. H. A. Examination of Electronic Structure Differences between CIGSSe and CZTSSe by Photoluminescence Study. J. Appl. Phys. 2015, 117, 235701 10.1063/1.4922493.

[ref26] Krustok J.; Schön J. H.; Collan H.; Yakushev M.; Mädasson J.; Bucher E. Origin of the Deep Center Photoluminescence in CuGaSe_2_ and CuInS_2_ Crystals. J. Appl. Phys. 1999, 86, 364–369. 10.1063/1.370739.

[ref27] Zacks E.; Halperin A. Dependence of the Peak Energy of the Pair-Photoluminescence Band on Excitation Intensity. Phys. Rev. B 1972, 6, 3072–3075. 10.1103/PhysRevB.6.3072.

[ref28] Rey G.; Larramona G.; Bourdais S.; Choné C.; Delatouche B.; Jacob A.; Dennler G.; Siebentritt S. On the Origin of Band-Tails in Kesterite. Sol. Energy Mater. Sol. Cells 2018, 179, 142–151. 10.1016/j.solmat.2017.11.005.

[ref29] Sarswat P. K.; Free M. L. A Study of Energy Band Gap versus Temperature for Cu_2_ZnSnS_4_ Thin Films. Phys. B Phys. Condens. Matter 2012, 407, 108–111. 10.1016/j.physb.2011.09.134.

[ref30] Levanyuk A. P.; Osipov V. V. Edge Luminescence of Direct-Gap Semiconductors. Sov. Phys. Uspekhi 1981, 24, 187–215. 10.1070/PU1981v024n03ABEH004770.

[ref31] Krustok J.; Collan H.; Hjelt K. Does the Low-Temperature Arrhenius Plot of the Photoluminescence Intensity in CdTe Point towards an Erroneous Activation Energy?. J. Appl. Phys. 1997, 81, 1442–1445. 10.1063/1.363903.

[ref32] Tiwari D.; Cattelan M.; Harniman R. L.; Sarua A.; Abbas A.; Bowers J. W.; Fox N. A.; Fermin D. J. Mapping Shunting Paths at the Surface of Cu_2_ZnSn(S, Se)_4_ Films via Energy-Filtered Photoemission Microscopy. iScience 2018, 9, 36–46. 10.1016/j.isci.2018.10.004. 30384132 PMC6215027

[ref33] Walter T.; Herberholz R.; Müller C.; Schock H. W. Determination of Defect Distributions from Admittance Measurements and Application to Cu(In, Ga)Se_2_ Based Heterojunctions. J. Appl. Phys. 1996, 80, 4411–4420. 10.1063/1.363401.

[ref34] Chen S.; Walsh A.; Gong X. G.; Wei S. H. Classification of Lattice Defects in the Kesterite Cu_2_ZnSnS_4_ and Cu_2_ZnSnSe_4_ Earth-Abundant Solar Cell Absorbers. Adv. Mater. 2013, 25, 1522–1539. 10.1002/adma.201203146. 23401176

[ref35] Han D.; Sun Y. Y.; Bang J.; Zhang Y. Y.; Sun H.; Li X.; Zhang S. B. Deep Electron Traps and Origin of P-Type Conductivity in the Earth-Abundant Solar-Cell Material Cu_2_ZnSnS_4_. Phys. Rev. B 2013, 87, 155206 10.1103/PhysRevB.87.155206.

