# Supplementary material for: Mapping the Energetics of Defect States in Cu2ZnSnS4 films and the Impact of Sb Doping
Source: ACS Appl Energy Mater. 2022 Mar 22;5(4):3933–40. doi: 10.1021/acsaem.1c03729 (PMC9044398; doi:10.1021/acsaem.1c03729)
Supplement: Supplementary file 1 — ae1c03729_si_001.pdf [file ae1c03729_si_001.pdf]

Supporting Information

Mapping the Energetics of Defect States in Cu_2ZnSnS_4 films and the Impact of Sb Doping

Devendra Tiwari[1,2,], Michael V Yakushev[3,4,5,6], Tristan Koehler[7], Mattia Cattelan[2,†]*

Neil Fox[2,8], Robert W Martin[3], Reiner Klenk[9] and David J Férmin[2,]*

[1]Department of Mathematics, Physics and Electrical Engineering, Northumbria University, Ellison Place, NE1 8ST Newcastle upon Tyne, United Kingdom

[2]School of Chemistry, University of Bristol, Cantocks Close, BS8 1TS Bristol, United Kingdom

[†]Dipartimento di Scienze Chimiche, Università degli Studi di Padova, Padova, Italy

[3]Department of Physics, SUPA, Strathclyde University, G4 0NG Glasgow, United Kingdom

[4]M.N. Miheev Institute of Metal Physics of the UB RAS, 18 S. Kovalevskoy St., 620108 Ekaterinburg, Russia

[5]Ural Federal University, 19 Mira St., 620002 Ekaterinburg, Russia

[6]Institute of Solid-State Chemistry of the UB RAS, 620990 Ekaterinburg, Russia

[7]Faculty of Physics, University of Duisburg-Essen, Forsthausweg 2, 47057 Duisburg, Germany

[8]H. H. Wills Physics Laboratory, University of Bristol, Tyndall Avenue, BS8 1TL Bristol, United Kingdom

[9]Helmholtz-Zentrum Berlin für Materialien und Energie, Hahn-Meitner-Platz 1, D-14109 Berlin, Germany

Email addresses for correspondence: devendra.tiwari@northumbria.ac.uk *and* David.Fermin@bristol.ac.uk

Contents

Figure S1: J-V Characteristics of no-dopants (ND), Sb-doped (Sb) and Na:Sb co-doped (Na:Sb) CZTS thin film solar cells under simulated AM 1.5G (100 mW/cm^2) illumination.

Figure S2: Spectral response of external quantum efficiency (EQE) of no-dopants (ND), Sb-doped (Sb) and Na:Sb co-doped (Na:Sb) CZTS thin film solar cells.

Figure S3: Estimation of bandgap energy from the EQE spectra.

Table S1: Statistical analysis of solar cell performance metrics.

Table S2: Summary of various defect states and optoelectronic parameters measured from EQE, admittance and PL spectroscopies.

Macroscopic device performance: J-V characteristics and quantum efficiency spectral response

Figure S1 and **S2** present the representative J-V characteristics under AM1.5 illumination and the spectral response of external quantum efficiency (EQE), contrasting the solar cell performance of devices incorporating no-dopants, Sb-doped and Na:Sb codoped CZTS films. The J-V curves clearly show a marked change in the V_{OC} and *FF*. Inset to **Figure S1** is a table of the performance parameters of solar cells. Similarly, EQE spectra (**Figure S2**) show a better charge collection at the longer wavelengths and a sharper CZTS absorption edge, indicating a lowering of the disorder in CZTS upon doping. Using the wavelength derivative of EQE in the neighbourhood of CZTS absorption, we calculate the bandgaps of the three CZTS layers (no-dopants and doped) to all be close to 1.4 eV (**Figure S3**) and to match the values obtained from diffuse reflectance spectroscopy in previous work.[1] As mentioned in the introduction, the kesterites suffer from a variety of forms of disorder which often result in tailing of the band edges.

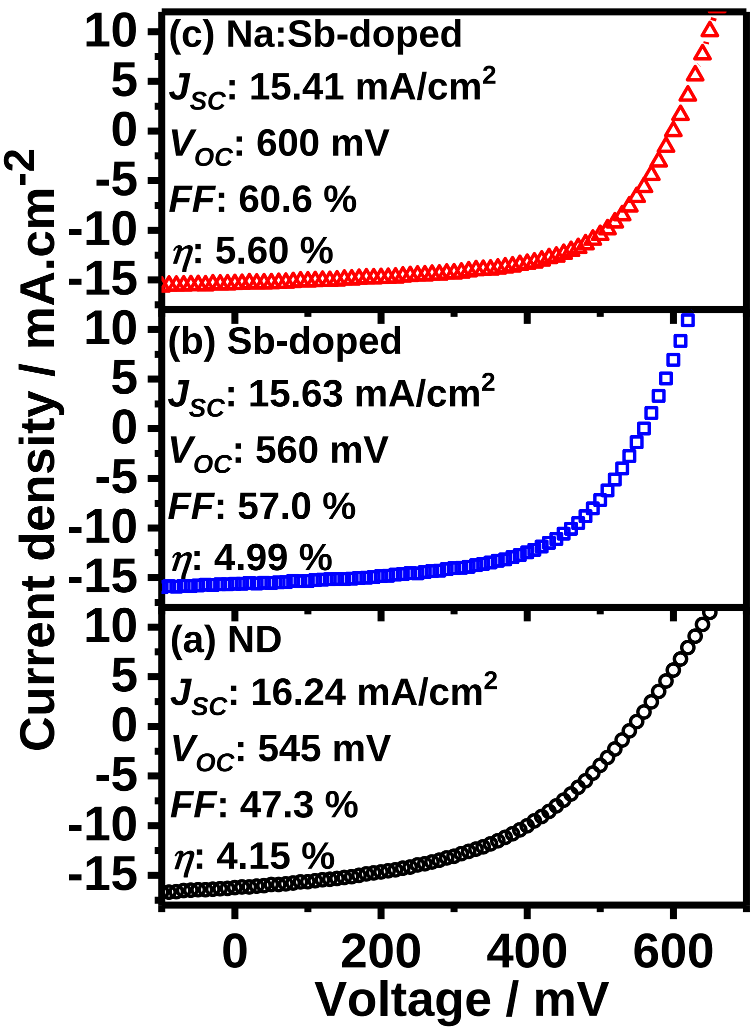

Figure S1: *J-V Characteristics of ND (a), Sb-doped (b) and Na:Sb-doped (c) CZTS solar cells under simulated AM 1.5G (100 mW/cm^2) illumination along with the performance metrics of short-circuit current density (J_{SC}), open-circuit voltage (V_{OC}), fill-factor (FF) and efficiency (η).*

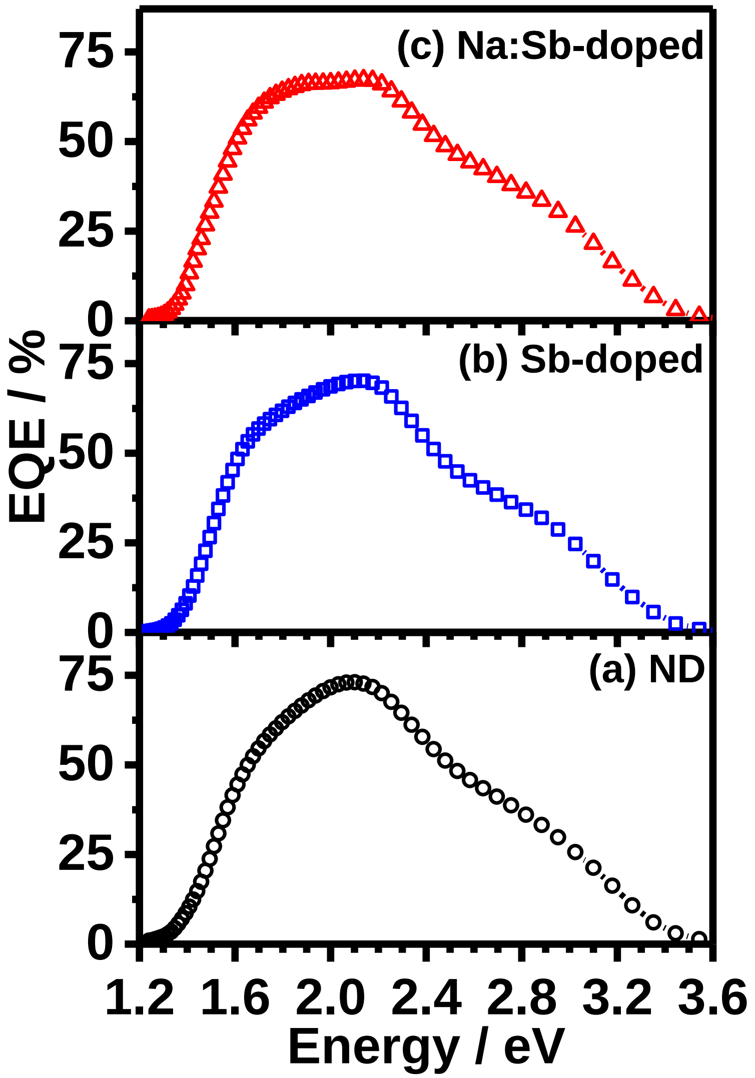

Figure S2: *Spectral response of external quantum efficiency (EQE) of ND (a), Sb-doped (b) and Na:Sb-doped (c) CZTS solar cells.*

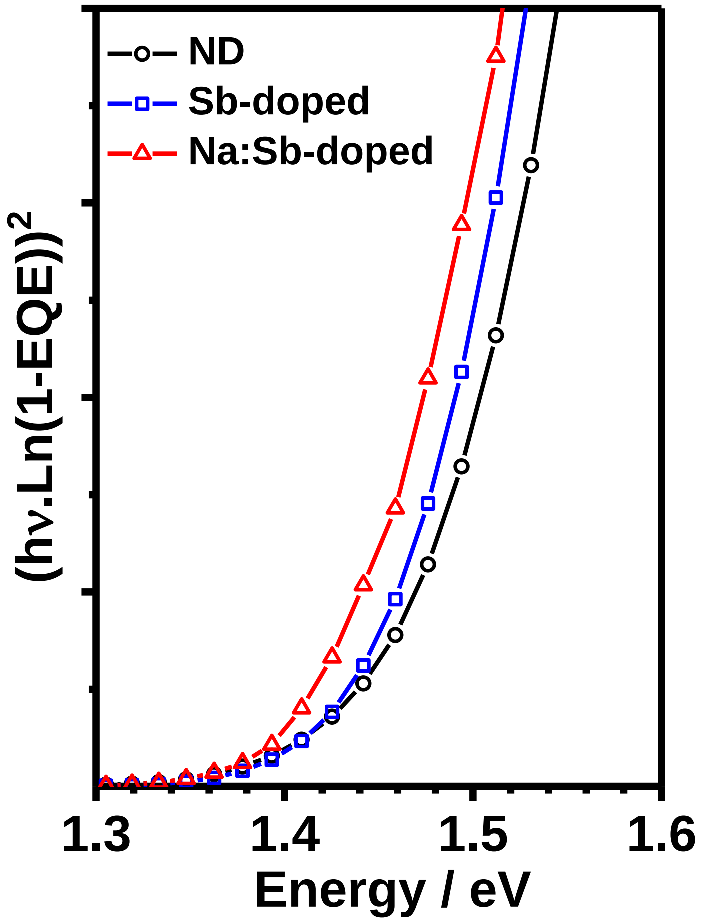

Figure S3: *Tauc-like plot to estimate the bandgap of CZTS from EQE data*

Table S1: *Statistical analysis of the performance metrics of solar cells based on ND, Sb-doped, Na:Sb-codoped CZTS. In total 216 devices were analysed including 72 devices for each type of sample*.

	**Average values of performance metrics ± standard deviation (Best value)**			
	V_{oc} / mV	J_{sc} / mA.cm^{-2}	*FF* / %	*Efficiency* / %
**ND**	492.09 ± 46.32 (550.29)	12.94 ± 2.03 (16.26)	42.77 ± 2.62 (47.10)	3.21 ± 0.64 (4.21)
**Sb-doped**	526.46 ± 43.54 (563.08)	15.03 ± 0.74 (15.25)	50.36 ± 3.14 (58.84)	4.70 ± 0.29 (5.05)
**Na:Sb-doped**	548.85 ± 27.55 (609.87)	15.48 ± 0.85 (14.89)	53.16 ± 2.55 (62.98)	5.04 ± 0.35 (5.72)

Table S2: *Summary of various defect states and optoelectronic parameters measured from EQE, admittance and PL spectroscopies. All the numbers are in the units of eV.*

**Method**	**Parameter**	**Assignment**	**ND**	**Sb-doped**	**Na:Sb-doped**
**Assumed**	VBM	VBM (eV)	0	0	0
**EQE**	CBM	CBM (eV)	1.41	1.41	1.41
**Admittance**	$E^1_{A,CfT}$		0.035	0.062	0.028
**Admittance**	$E^2_{A,CfT}$	$E_A - E_V$ (eV)	0.106	0.236	0.125
**Admittance**	$E^3_{A,CfT}$		0.359	-	-
**PL**	W_1 or $E^{1,Sb}_{A,PL}$	Γ (eV)	0.035	0.018	0.022
**PL**	$E^2_{A,PL}$	$E_C - E_D$ (eV)	0.121	0.077	0.126
**PL**	E_{Peak}	Peak position (eV)	1.15	1.06	1.17
**PL Admittance**	$E_{Peak} + (E_A + E_D) + 2\Gamma$	QDAP (eV)	1.45	1.41	1.43
**EQE, diffuse reflectance**	Band gap	E_g (eV)	1.41	1.41	1.41

Reference

(1) Tiwari, D.; Koehler, T.; Lin, X.; Harniman, R.; Griffiths, I.; Wang, L.; Cherns, D.; Klenk, R.; Fermin, D. J. Cu_2ZnSnS_4 Thin-Films Generated from a Single Solution Based Precursor: The Effect of Na and Sb Doping. *Chem. Mater.* **2016**, *28*, 4991–4997.
